# Insomnia and elevated risk of major depressive disorder in older adult, long-term breast cancer survivors vs a matched cohort

**DOI:** 10.1093/sleep/zsaf322

**Published:** 2025-10-10

**Authors:** Michael R Irwin, Richard Olmstead, LieHong Chen, Reina Haque

**Affiliations:** Cousins Center for Psychoneuroimmunology, Semel Institute for Neuroscience and Human Behavior, University of California, Los Angeles, CA, USA; Department of Psychiatry and Biobehavioral Sciences, David Geffen School of Medicine, University of California, Los Angeles, CA, USA; Cousins Center for Psychoneuroimmunology, Semel Institute for Neuroscience and Human Behavior, University of California, Los Angeles, CA, USA; Department of Psychiatry and Biobehavioral Sciences, David Geffen School of Medicine, University of California, Los Angeles, CA, USA; Department of Research & Evaluation, Kaiser Permanente Southern California, Pasadena, CA, USA; Department of Research & Evaluation, Kaiser Permanente Southern California, Pasadena, CA, USA; Department of Health Systems Science, Kaiser Permanente Bernard J. Tyson School of Medicine, Pasadena, CA, USA

**Keywords:** breast cancer, survivorship, depression, insomnia

## Abstract

**Study Objectives:**

Depression frequently occurs after diagnosis of breast cancer diagnosis. This study examines the incidence of depression and whether insomnia exaggerates depression risk in long-term breast cancer survivors (BCSs).

**Methods:**

A sample of 636 nondepressed females, aged 55 to 85 years, was recruited between 2015 and 2020 from a diverse community-based health plan; 315 were BCSs at least 2 years postdiagnosis, and 321 were an aged-matched comparison cohort. Both groups were stratified by the presence or absence of insomnia at baseline. The primary outcome, incident and recurrent major depressive disorder, was diagnosed over 32 months. Cox proportional hazards models estimated risk of depression (hazard ratio [HR], 95% confidence interval [CI]).

**Results:**

A total of 310 (98.4%) BCSs, and 309 (96.3%) comparisons completed 32 months follow-up. Relative to the comparisons, risk of depression was elevated in BCSs (HR = 5.94; 95% CI = 3.34% to 10.54%, *p* < .001). Insomnia, as defined by the Insomnia Severity Index (>8), further increased depression risk BCS (HR = 9.91; 95% CI = 4.92% to 19.96%; *p* < .001), but not in the comparisons.

**Conclusions:**

Long-term BCSs have a heightened risk of major depressive disorder, and even subthreshold insomnia exaggerates that risk. Given that insomnia treatment can effectively prevent depression, insomnia screening and treatment have implications for depression prevention in BCSs.

**Statement of Significance**

Breast cancer survivors with insomnia show a markedly elevated risk of depression, which supports the urgent need to screen for insomnia in cancer survivors and to develop and implement insomnia treatment to prevent depression in this high-risk population.

## Graphical Abstract

**Graphical Abstract:**
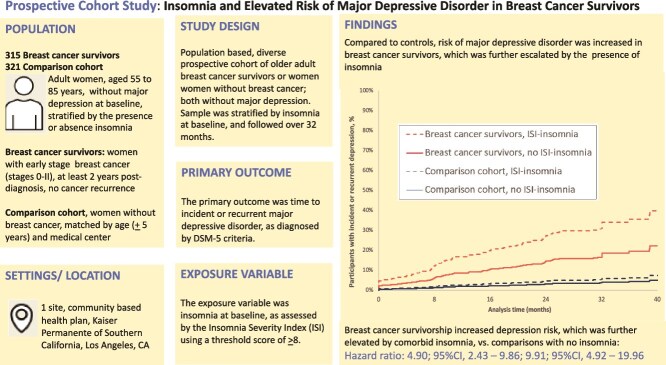

## Introduction

Depression prevalence exceeds 25% in breast cancer survivors (BCSs), higher than other populations with cancer [1–7]. Depression is a significant risk factor for cognitive decline, disability, comorbidities, and all-cause mortality [8, 9], and importantly is a predictor of tumor progression and cancer mortality in older adult cancer survivors [10, 11]. Whereas it is known that depression arises most frequently in the first year after diagnosis and treatment of breast cancer [12, 13], no prospective study, to our knowledge, has evaluated whether incident or recurrent depression is elevated in long-term BCSs as compared to women without cancer. Moreover, there is an absence of research data in older cancer survivors, which is striking given that older adults account for nearly 20% of the population in the United States, comprise 67% of cancer survivors [14], are most vulnerable to depression burden, and report the lowest use of mental health services [15]. Moreover, a key uncertainty is whether an increased risk of depression in cancer survivors might be due to modifiable risk factors.

In BCSs, insomnia is a prominent behavioral symptom with an estimated 60% reporting clinically significant insomnia symptoms [16–18], which is twice the prevalence in the general population [16, 19, 20]. Moreover, insomnia persists over the long term during survivorship [21]. In adults without cancer, insomnia is a robust predictor of major depressive disorder (i.e. depression) [22], contributing to a twofold greater risk of depression, independent of other known risk factors including prior history of depression, other depressive symptoms, and use of antidepressant medications [23]. However, there is a striking absence of research that has examined whether insomnia exaggerates depression risk in long-term cancer survivors. Given that insomnia treatment such as cognitive behavioral therapy for insomnia (CBT-I) can prevent depression and reduce the likelihood of depression by over 50% in older adults [24], understanding the association between insomnia and depression risk in cancer survivors could substantially alter clinical management of cancer survivorship and lead to the successful planning, testing, and implementation of depression prevention interventions.

To address these gaps, this prospective cohort study determined the association between insomnia and the risk of incident and recurrent major depressive disorder, as defined by *Diagnostic and Statistical Manual (DSM)-5* criteria [25], in older adult BCSs with minimal depressive symptoms and without current depression, as compared to an aged-matched comparison cohort (CC), over a 32 month follow-up. We hypothesized that major depressive disorder would be more likely to occur in long-term BCSs than in the cohort without cancer. Moreover, we hypothesized that insomnia would exaggerate risk of depression in BCSs.

## Materials and Methods

### Study design

The Sleep to Thrive Study is a population-based, diverse prospective cohort study of 315 older adult, BCSs without current depression, and 321 older adult women without breast cancer or depression (i.e. CC), who were followed over 32 months to ascertain the incidence of major depressive disorder (depression). Study recruitment occurred between July 1, 2015 and June 30, 2020, with protocol modification in July 2016 to extend follow-up from 24 to 32 months. Follow-up was completed July 2023. Logistic challenges during the COVID 19 pandemic delayed endpoint follow-up up to 8 months for some participants.

The study was reviewed and approved by the institutional review boards of the University of California, Los Angeles (UCLA) and by Kaiser Permanente of Southern California (KPSC). Written informed consent was obtained from all participants. All data were de-identified. The study followed the Strengthening the Reporting of Observational Studies in Epidemiology reporting guidelines [26].

### Participants

Participants were identified from KPSC, a large nonprofit health plan that serves over 4.8 million members. BCSs were identified using the KPSC Surveillance Endpoints & End Results (SEER)-affiliated cancer registry [27]. Eligibility criteria were early-stage breast cancer (stages 0–II); at least 2 years postdiagnosis; no recurrence; female sex; 55 to 85 years old; residing in Los Angeles County; absence of major depressive disorder, or other major psychiatric disorder within the last year as identified by the Structured Clinical Interview for Diagnosis (SCID) [28] using DSM-5 criteria [25]; and absence of inflammatory-related medical condition as identified by electronic medical records and Charlson Comorbidity Index [29, 30]; examples of excluded inflammatory conditions are rheumatoid arthritis, psoriasis, and systemic lupus erythematosus. Additionally, participants underwent screening for the exclusion of sleep apnea using a questionnaire approach (i.e. Berlin Questionnaire for Sleep Apnea) [31]; those who screened positive or scored as “high risk” for sleep apnea were excluded. The CC included eligible females without a history of breast cancer, who were randomly identified from KPSC records, matched by age (±5 years) and medical center.

### Procedures

Data were collected in five waves by trained master’s level research associates over 8 month intervals. Interview assessments were in-person or telephone-based. Electronic medical records were monitored between follow-up intervals to identify depression episodes. Interviewers were blinded to baseline exposure data.

### Primary outcome

The primary outcome was time to incident or recurrent major depressive disorder as diagnosed by *DSM-5* criteria [25]. A depression event was the first day of the episode after baseline, using clinical data from the SCID or electronic medical records. Assignment of diagnosis was made in a consensus meeting blinded to other data. Incident or recurrent major depressive disorder was assigned based on prior depression history and analyzed as a composite variable.

### Exposure variable

Insomnia at baseline was the exposure variable, as assessed by the Insomnia Severity Index (ISI) using a threshold score of *>*8. Hence, the exposure variable is named ISI-insomnia.

The ISI score *>*8 has 99.4% sensitivity and 91.8% specificity relative to the diagnosis of insomnia by a structured interview using DSM-IV criteria [32]. The ISI was used, because this 7-item screening tool can be readily implemented in clinical oncology settings. DSM-5 history of insomnia is a secondary exposure.

### Baseline covariates

In our conceptual model, we examined a comprehensive set of covariates that included clinical, psychosocial, and sociodemographic factors deemed important in the literature. The selection of covariates to be collected was guided by meta-analytic evidence that identified risk factors for depression in older adults [33]. In addition to female sex and sleep disturbance, meta-analysis found that medical disability and prior depression were significantly associated with depression, with nonsignificant effects for less education and poor social support [33]. In addition to these variables, sociodemographic characteristics such as age, self-reported race/ethnicity using National Institutes of Health defined categories, education, income, and employment status were assessed. Other relevant variables related to insomnia were evaluated including body mass index (BMI) and vasomotor symptoms [34]. Additional depression-specific variables that have been related to risk of depression in older adults [22] were also evaluated, including lifetime history of depressive disorder, insomnia, or alcohol use disorder [28]; current use of antidepressant medications; and severity of depressive symptoms with Inventory for Depressive Symptoms-Self Report (IDS-SR) [35].

Unlike previous studies that relied on self-report, medical comorbidities were identified using electronic medical records with scoring calculated by the Charlson Comorbidity Index [29, 30]; the Charlson Comorbidity Index includes 17 categories of comorbidity, each with an assigned score of 1–6, depending on the risk of death associated with the condition; maximum score is 29. Social support, as indexed by the number of close friends, was evaluated by the Interpersonal Support Evaluation List [36]. To assess the history of depression, insomnia, or alcohol use disorder, the Structured Clinical Interview for Diagnostic and Statistical Manual-5 (SCID-5) was administered. SCID-5 confirmed absence of DSM-5 major depressive disorder at entry for at least 1 year, as ascertained by absence or presence of lifetime DSM-5 major depressive disorder. Depressive symptom severity was evaluated using the IDS-SR. This scale includes 30 items; each item has four statements that reflect various degrees of symptom severity, scored on a four-point scale from 0 to 3. Four items related to insomnia symptoms were removed, yielding 26 items and range from 0 to 78. Antidepressant medication use was obtained from the electronic medical records and included only such use for the treatment of depression; use of mirtazapine, often used for the treatment of depression and insomnia, was infrequent. As noted, insomnia severity was assessed using the ISI, which rates the severity of sleep disturbance according to the International Classification of Sleep Disorders (Second Edition) for insomnia diagnosis and the DSM-5; the maximum score of 28. Scores below 8 on the ISI indicate the absence of insomnia [32]. Cancer-specific variables included time since breast cancer diagnosis, stage, and adjuvant therapies as extracted from the KPSC-SEER-affiliated cancer registry.

### Sample size

Sample sizes were determined for main effects of group and insomnia status, with power set at *α* = 0.05 and a minimum power of 80% for the expected unbalanced comparison estimating depression risk in groups stratified by insomnia. The annual incidence of depression is between 3.0% and 4.0% [23, 37]; insomnia was assumed to predict a twofold greater risk of depression [23]. Over 32 months, 20% attrition was estimated. For 80% power, 140 participants with insomnia would be required. It was estimated that 30% of BCSs, and 15% of comparisons would have insomnia. Hence, the breast cancer sample size was *n* = 310. With an equal size for the CC, there was 95% power to detect a twofold increase in depression risk between groups.

### Statistical analysis

Standard descriptive statistics were used to characterize the two groups. Person-year rates of the primary outcome, incident and recurrent depression, were calculated for both groups. For the time to event analyses using multivariable Cox proportional hazards regression models, participants were followed to depression event, death, disenrollment from the study, or completion of 32-month follow-up, whichever occurred first. For those who disenrolled or completed without a depression event, cases were censored to the last follow-up. Analyses first evaluated depression events as a function of group status. Next, categorical ISI-insomnia status was tested as the predictor of depression. Finally, a four-group model was tested; this model crossed group and insomnia categories to test whether group and ISI-insomnia were additive. Sensitivity analyses were performed with a nested set of sequential models, in which variables that either differed between groups and/or were associated with depression risk were added [33]. Secondary analyses estimated the risk of depression as a function of DSM-5 insomnia history. The hazard ratio (HR) and 95% confidence interval (CI) were calculated using Cox proportional hazards regression models. The proportional hazards assumptions were tested using Schoenfeld residuals before and after adjustment. SAS 9.4 and SPSS (Version 29) were used for data management and analyses.

## Results

### Participants

A total of 636 participants were enrolled; 315 BCSs and 321 CC. Compared to the CC, BCSs were 2 years younger, reported less education and less income, were less likely to be employed, and were more likely to have medical comorbidities, depression history, current insomnia, and insomnia history. Severity of depressive symptoms was similarly low in both groups (Table 1). In the BCSs, cancer-specific characteristics included time since diagnosis (mean *y ±* SD; 6.3 + 3.9); stage at diagnosis (no. %; Stage 0 = 61, 19.4%; Stage I = 150, 47.6%; Stage II = 104, 33.0%); and cancer treatment (no., %; radiation, 166, 52.7%; hormonal, 161, 51.1%; chemotherapy, 93 29.5%).

**Table 1:** Clinical and demographic characteristics of study participants at baseline^*^

Characteristic	Breast cancer survivors (*n* = 315)	Comparison cohort (*n* = 321)	Statistic (*df*); *p*
Age, mean (SD), y	70.8 (6.4)	72.7 (5.8)	*t*(634) = 4.03; *p* < .001
Race^†^			*Χ* ^2^(3) = 6.72; *p* = .082
American Indian/Alaskan Native	0 (0%)	0 (0%)	
Asian	28 (8.9%)	20 (6.2%)	
Native Hawaiian/Pacific Islander	0 (0%)	4 (1.2%)	
Black/African-American	66 (21.0%)	57 (17.8%)	
White	221 (70.2%)	240 (74.8%)	
More than one	0 (0%)	0 (0%)	
Ethnicity^†^			*Χ* ^2^(1) = 0.69; *p* = .41
Hispanic/Latino	4 (1.3%)	2 (0.6%)	
Not Hispanic/Latino	314 (98.7%)	319 (99.4%)	
Education, mean (SD), y	15.8 (2.9)	16.3 (2.6)	*t*(590) = 2.18; *p* = .015
Income, mean (SD), $1000	80.6 (49.2)	90.7 (49.9)	*t*(599) = 2.51; .013
Employment status			*Χ* ^2^(1) = 3.57; .059
No	204 (66.0%)	233 (73.3%)	
Yes	105 (34.0%)	85 (26.7%)	
Medical comorbidity^‡^			*Χ* ^2^(2) = 57.8; *p* < .001
0	87 (27.6%)	163 (50.8%)	
1–2	125 (39.7%)	124 (38.6%)	
3+	103 (32.7%)	34 (10.6%)	
Social Support			
Number of close friends	7.4 (5.4)	8.0 (7.4)	*t*(619) = 1.18; .12
Vasomotor symptoms			*Χ* ^2^(1) = 16.7; *p* < .001
No	136 (44.4%)	89 (28.6%)	
Yes	170 (55.6%)	222 (71.4%)	
Body mass index, mean (SD)^§^	26.8 (4.1)	25.3 (3.9)	*t*(573) = 4.76; *p* < .001
DSM-5 alcohol abuse history			*Χ* ^2^(1) = 0.49; .48
No	295 (96.7%)	304 (95.3%)	
Yes	10 (3.3%)	15 (4.7%)	
DSM-5 depression history			*Χ* ^2^(1) = 41.5; *p* < .001
No	212 (67.3%)	284 (88.5%)	
Yes	103 (32.7%)	37 (11.5%)	
Depressive symptoms, mean (SD)	7.4 (6.8)	6.5 (5.7)	*t*(626) = 1.74; *p* = .082
Depressive symptoms, categorical			*Χ* ^2^(2) = 5.53; *p* = .063
0–11 (None)	241 (78.2%)	273 (85.3%)	
12–22 (Mild)	53 (17.22%)	39 (12.2%)	
23–33 (Moderate)	14 (4.5%)	8 (2.5%)	
Antidepressant medication use			*Χ* ^2^(1) = 7.45; *p* = .006
No	272 (89.8%)	257 (82.1%)	
Yes	31 (10.2%)	56 (17.9%)	
DSM-5 insomnia history			*Χ* ^2^(1) = 11.2; *p* < .001
No	219 (69.5%)	259 (80.7%)	
Yes	96 (30.5%)	62 (19.3%)	
Insomnia severity, mean (SD)	5.9 (5.4)	4.6 (4.9)	*t*(634) = 3.30; *p* < .001
Insomnia severity, categorical			*Χ* ^2^(1) = 16.1; *p* < .001
<8, no insomnia	202 (64.1%)	252 (78.5%)	
≥8, insomnia	113 (35.9%)	69 (21.5%)	
Sleep medication use			*Χ* ^2^(1) = 1.29; *p* = .29
No	217 (70.0%)	237 (74.1%)	
Yes	93 (30.0%)	83 (25.9%)	

^*^Data are presented as number (percentage) unless otherwise indicated.

^†^Race and ethnicity were reported by the participants.

^‡^Medical comorbidity was evaluated using the Charlson Comorbidity Index.

§ BMI was calculated as weight in kilograms divided by height in meters squared.

### Follow-up retention

**Figure 1:**
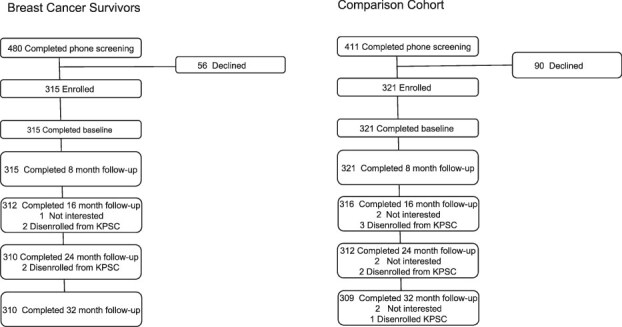
Participant flowchart. Details regarding screening, eligibility assessment, and follow-up are provided in the *Materials and Methods* section.

Follow-up retention was high; 98.4% (*n* = 310/315) of BCSs and 96.3% (*n* = 309/321) of CC completed 32 month follow-up assessment (*χ*^2^(1) = 2.83; *p* = .10; Figure 1).

### Primary outcome: breast cancer survivors vs comparison cohort

The primary outcome, DSM-5 incident or recurrent major depressive disorder, occurred in 77 BCSs (24.4% of the sample; 10.0 events/100 person years; 95% CI = 6.66% to 13.27%; 10.0% annual incidence) and 14 of CC (4.4 % of the sample; 1.7 events/100 person years; 95% CI = 0.28% to 3.11%; 1.7% annual incidence; χ^2^(1) = 52.3; *p* < .001).

**Figure 2:**
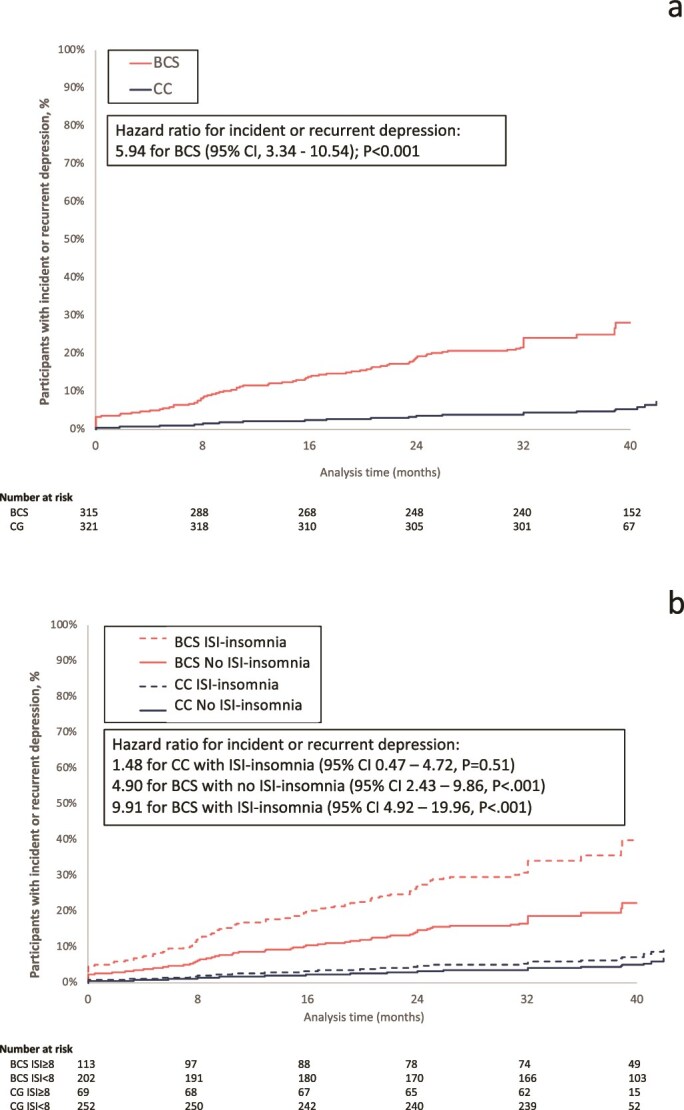
Time to incident or recurrent major depressive disorder depression event by group (a), and by group stratified by insomnia (b) at baseline. Panel A: Cumulative incidence of major depressive disorder among BCSs and participants in the CC. Panel B: Cumulative incidence of major depressive disorder BCSs with insomnia (BCS Insomnia); BCSs without insomnia (BCS No Insomnia); CC with insomnia (CC Insomnia); CC without insomnia (CC No Insomnia) Groups were BCSs and CC. Participants were followed up for a 32 month assessment, which was delayed to 40 months for some participants during the COVID 19 pandemic.

The risk of depression was nearly sixfold greater in BCSs vs CC (HR = 5.94; 95% CI = 3.34% to 10.54%, *p* < .001; Figure 2, A; Table 2, Model 0). After adjusting for age, education, comorbidities, and vasomotor symptoms, risk of depression was fivefold greater in BCSs, (HR = 5.26; 95% CI = 3.05% to 10.38%, *p* < .001; Table 2, Model 1), with similar effects after adjusting for depression history, depressive symptoms, and antidepressant medication use (HR = 4.65; 95% CI = 2.57% to 9.78%; *p* < .001; Table 2, Model 2). Risk of depression increased precipitously in BCSs vs CC over follow-up. Other variables that differed between groups, including income, and employment status, were not related to depression risk.

**Table 2:** Prospective associations between breast cancer survivorship, current ISI-insomnia, and their interaction with incident or recurrent DSM-5 major depressive disorder over 32 months follow-up^*^

	**Model 0**	**Model 1**	**Model 2**
**Group**			
Comparison cohort	1.00 (REF)	1.00 (REF)	1.00 (REF)
Breast cancer survivors	5.94 (3.34–10.54) < .001	5.26 (2.85–9.71) < .001	4.65 (2.45–8.83) < .001
Age (per year)	–	1.00 (0.97–1.04) .90	1.02 (0.98–1.06) .28
Education (per year)	–	0.96 (0.89–1.04) .28	0.92 (0.85–1.01) .08
Medical comorbidity (per group)	–	1.07 (0.80–1.44) .65	1.08 (0.78–1.49) .65
Vasomotor symptoms (vs none)	–	1.34 (0.84–2.14) .23	0.99 (0.61–1.62) .99
Depression history (vs none)	–	–	4.35 (2.49–7.60) < .001
Depressive symptoms (per group)	–	–	1.51 (1.01–2.26) .043
Antidepressant medication use (vs none)	–	–	4.45 (2.60–7.60) < .001
**ISI-Insomnia**			
No	1.00 (REF)	1.00 (REF)	1.00 (REF)
Yes	2.35 (1.56–3.55) < .001	2.01 (1.28–3.17) .003	1.20 (0.71–2.03) .51
Age (per year)		0.98 (0.95–1.02) .35	1.00 (0.96–1.04) .89
Education (per year)		0.95 (0.88–1.03) .23	0.94 (0.86–1.01) .11
Medical comorbidity (per group)		1.41 (1.06–1.89) .020	1.31 (0.94–1.79) .11
Vasomotor symptoms (vs none)		0.97 (0.61–1.54) .90	0.88 (0.54–1.43) .62
Depression history (vs none)			6.22 (3.66–10.56) < .001
Depressive symptoms (per group)			1.61 (1.08–2.42) .021
Antidepressant medication use (vs none)			3.02 (1.84–4.95) < .001
**Group X ISI-Insomnia**			
Comparison cohort, no insomnia	1.00 (REF)	1.00 (REF)	1.00 (REF)
Comparison cohort, insomnia	1.48^1^ (0.47–4.72) .51	1.33^1^ (0.41–4.26) .64	0.95^1^ (0.28–3.18) .93
Breast cancer survivors, no insomnia	4.90^2^ (2.43–9.86) < .001	4.43^2^ (2.13–9.22) < .001	4.28^2^ (2.00–9.17) < .001
Breast cancer survivors, insomnia	9.91^3^ (4.92–19.96) < .001	8.17^3^ (3.87–17.24) < .001	5.13^2^ (2.23–11.80) < .001
Age (per year)	–	1.01 (0.97–1.05) .69	1.02 (0.98–1.07) .26
Education (per year)	–	0.97 (0.89–1.04) .38	.093 (0.86–1.01) .10
Medical comorbidity (per group)	–	1.09 (0.81–1.46) .58	1.08 (0.78–1.49) .65
Vasomotor symptoms (vs none)	–	1.28 (0.80–2.04) .31	0.98 (0.60–1.59) .93
Depression history (vs none)	–	–	4.30 (2.46–7.52) < .001
Depressive symptoms (per group)	–	–	1.46 (0.94–2.24) .09
Antidepressant medication use (vs none)	–	–	4.47 (2.62–7.64) < .001

^*^Data are displayed as HR, 95% CIs, and *p*-values.

Groups that differ by numbered superscript are significantly different at *p* < .02.

### Primary outcome: breast cancer survivors vs comparison cohort, by insomnia status

In the overall sample, risk of depression was over twofold greater in those who reported ISI-insomnia, as compared to those without ISI-insomnia (HR = 2.35; 95% CI = 1.56% to 3.55%; *p* < .001). When groups were stratified by ISI-insomnia, depression occurred in 38 BCSs with ISI-insomnia (33.6% of the group; 14.5 events/100 person years; 95% CI = 7.94% to 21.11%; 14.5 % annual incidence); 39 BCSs without ISI-insomnia (19.3% of the group; 7.3 = events/100 person years; 95% CI = 3.70% to 10.92%; 7.3% annual incidence), 4 comparisons with ISI-insomnia (5.8% of the group; 2.3 events/100 person years; 95% CI = 0% to 5.87%; 2.3% annual incidence); and 10 comparisons without ISI-insomnia (4.0% of the group; 1.6 events/100 person years; 95% CI = 0.02% to 3.11%; 1.6% annual incidence). Annual incidence differed across the four groups (*χ*^2^(3) = 64.6; *p* < .001).

Compared to CC without ISI-insomnia, risk of depression was fivefold greater in BCSs without ISI-insomnia (HR = 4.90; 95% CI = 2.43% to 9.86%; *p* < .001), and over ninefold greater in BCSs with ISI-insomnia (HR = 9.91; 95% CI = 4.92% to 19.96%; *p* < .001; Table 2, Model 0), significantly different than all other groups (*p* < .01). Risk of depression was not significantly elevated in the CC with ISI-insomnia (HR = 1.48; 95% CI = 0.41% to 4.26%; *p* = .64; Table 2, Model 0). Adjustment for demographic and clinical variables (Table 2, Model 1) and for depression-specific variables yielded similar results (Table 2, Model 2). The test for the proportional hazard assumption with Schoenfeld residuals gave a *p* > .07 or more across all covariates, indicating no violations. In sum, the risk of depression increased steeply in the BCSs, and ISI-insomnia interacted with breast cancer status to exaggerate that risk (Figure 2, B).

### Secondary analyses

The secondary exposure variable was history of DSM-5 insomnia disorder. Insomnia history was associated with an almost fourfold greater likelihood of depression (HR = 3.8; 95% CI = 2.52% to 5.75%; *p* < .001) (Table 3, Model 0). Compared to comparisons without insomnia, risk of depression was 25-fold greater in BCSs with insomnia history (HR = 25.9; 95% CI = 10.16% to 65.60%; *p* < .001), significantly greater than the risk in BCSs without insomnia history (HR = 9.0; 95% CI = 3.54% to 22.99%; *p* < .001), and in the CC with insomnia history (HR = 7.6; 95% CI = 2.55% to 22.70%; *p* < .001; Table 3; Model 0), with similar results in adjusted analyses (Table 3, Models 1–2).

**Table 3:** Prospective associations between breast cancer survivorship, DSM-5 insomnia history, and their interaction with incident or recurrent DSM-5 major depressive disorder over 32 months of follow-up^*^

	**Model 0**	**Model 1**	**Model 2**
**Group**			
Comparison cohort	1.00 (REF)	1.00 (REF)	1.00 (REF)
Breast cancer survivors	5.94 (3.34–10.54) < .001	5.26 (2.85–9.71) < .001	4.65 (2.45–8.83) < .001
Age (per year)	–	1.00 (0.97–1.04) .90	1.02 (0.98–1.06) .28
Education (per year)	–	0.96 (0.89–1.04) .28	0.92 (0.85–1.01) .08
Medical comorbidity (per group)	–	1.07 (0.80–1.44) .65	1.08 (0.78–1.49) .65
Vasomotor symptoms (vs none)	–	1.34 (0.84–2.14) .23	0.99 (0.61–1.62) .99
Depression history (vs none)	–	–	4.35 (2.49–7.60) < .001
Depressive symptoms (per group)	–	–	1.51 (1.01–2.26) .04
Antidepressant medication use (vs none)	–	–	4.45 (2.60–7.60) < .001
**DSM-5 Insomnia History**			
No	1.00 (REF)	1.00 (REF)	1.00 (REF)
Yes	3.81 (2.52–5.75) < .001	3.63 (2.32–5.67) < .001	1.71 (1.04–2.82) .04
Age (per year)	–	0.99 (0.95–1.02) .42	1.00 (0.97–1.04) .86
Education (per year)	–	0.96 (0.88–1.04) .28	0.95 (0.87–1.03) .20
Medical comorbidity (per group)	–	1.52 (1.12–2.04) .007	1.31 (0.95–1.79) .10
Vasomotor symptoms (vs none)	–	1.09 (0.69–1.72) .73	0.91 (0.56–1.46) .69
Depression history (vs none)	–	–	5.72 (3.34–9.81) < .001
Depressive symptoms (per group)	–	–	1.55 (1.06–2.26) .03
Antidepressant Medication Use (vs none)	–	–	2.74 (1.65–4.53) < .001
**Group X DSM-5 Insomnia History**			
Comparison cohort, no insomnia history	1.00 (REF)	1.00 (REF)	1.00 (REF)
Comparison cohort, insomnia history	7.61^2^ (2.55–22.70) < .001	7.30^2^ (2.44–21.86) < .001	4.06^2^ (1.34–12.30) .01
Breast cancer survivors, no insomnia history	9.02^2^ (3.54–22.99) < .001	8.01^2^ (3.05–21.00) < .001	8.49^2^ (3.15–22.42) < .001
Breast cancer survivors, insomnia history	25.87^3^ (10.16–65.90) < .001	22.23^3^ (8.46–58.40) < .001	10.87^2^ (3.64–27.76) < .001
Age (per year)	–	1.01 (0.97–1.04) .69	1.02 (0.98–1.07) .24
Education (per year)	–	0.97 (0.89–1.05) .48	.094 (0.86–1.02) .12
Medical comorbidity (per group)	–	1.17 (0.87–1.59) .31	1.09 (0.79–1.50) .62
Vasomotor symptoms (vs none)	–	1.39 (0.87–2.22) .18	0.98 (0.60–1.60) .95
Depression History (vs none)	–	–	4.01 (2.28–7.03) < .001
Depressive symptoms (per group)	–	–	1.44 (0.95–2.19) .09
Antidepressant medication use (vs none)	–	–	4.13 (2.37–7.22) < .001

^*^Groups that differ by numbered superscript are significantly different at *p* < .01.

In sensitivity analyses in BCSs, neither time since breast cancer diagnosis, stage of cancer (stages 0–II), nor adjuvant treatment (i.e. radiation, hormonal, chemotherapy) was related to depression risk (all *p*’s > .16).

### Adverse events

Adverse events were actively monitored during follow-up at each assessment interval, and no adverse events were identified. No participant, including those with an incident major depression, experienced suicidal symptoms that met the threshold for clinical referral.

## Discussion

The results of this prospective study confirmed our study hypotheses. First, BCSs showed a marked fivefold increase in the risk of DSM-5 diagnostically defined, incident and recurrent major depressive disorder, as compared to the matched CC. Second, relative to the CC without ISI-insomnia, BCSs with ISI-insomnia showed an over ninefold higher risk of depression, whereas matched comparison with ISI-insomnia did not show an elevated risk of depression. The annual incidence of depression exceeded 14% in BCSs with ISI-insomnia, more than double the rate in BCSs without ISI-insomnia, and sevenfold greater than the CC, which did not differ between those with and without ISI-insomnia. The history of diagnostic insomnia disorder was also found to predict depression risk, similar to the effects of current ISI-insomnia. Importantly, these findings remained robust, even after adjusting for demographic characteristics, comorbidities, lifetime history of depression, other depressive symptoms, and antidepressant use. With a greater than 95% rate of follow-up over 32 months, this prospective study is the first, to our knowledge, to show that depression risk is markedly elevated in older adult, long-term BCSs as compared to a noncancer CC. Moreover, the presence of ISI-insomnia, as well as insomnia history, further exaggerates depression risk in BCSs. The BCSs, who were on average 6 years after diagnosis and treatment, were beyond the acute period of vulnerability to depression [13, 38].

Screening for insomnia using well-validated tools, as recommended by the National Comprehensive Cancer Network’s Survivorship Guideline [39], is urgently needed for cancer survivors [18]. Yet, less than 50% of survivorship programs in NCI-designated comprehensive cancer centers routinely screen for insomnia or sleep disorders [40]. Furthermore, it is reported that only about a third of oncologists, and less than half of oncology nurses, ask about sleep [41, 42]. The lack of attention to insomnia in cancer survivors is striking, given its prevalence [42] and the robust efficacy of nonpharmacologic treatments such as CBT-I to treat insomnia in cancer survivors [43] and adults [44]. Further, scalable and community accessible treatments, such as Tai Chi and mindfulness meditation, are also available, both of which are noninferior to CBT-I in yielding robust and sustained insomnia remission in cancer survivors [45, 46]. The reasons for the low rate of screening for insomnia are not known, although it is possible that healthcare settings may lack resources to screen for sleep problems in cancer survivors. Further, constraints such as prioritizing cancer care, the lack of training to identify and treat sleep problems among oncologists, limited access to specialized providers to address sleep problems, or lack of insurance coverage for evidence-based sleep treatments may all contribute to this issue.

In cancer survivors, especially BCSs, identification and treatment of insomnia can reduce the burden of insomnia and might also serve as an effective strategy to prevent depression. Indeed, in healthy older adults with insomnia, we demonstrated that 2 months of CBT-I, the recommended first line treatment of insomnia [47], resulted in a 50% lower likelihood of incident and recurrent major depressive disorder during 36 months of follow-up [24]. This selective prevention strategy was efficient, with a number needed to treat (NNT) of 7 to prevent major depressive disorder [24]; NNT is an estimate (i.e. average number) of participants who need to be treated with CBT-I to prevent one additional episode of one major depressive disorder. Because NNT is defined as inverse of the absolute risk reduction, in which both risk of depression and the efficacy of CBT-I is taken into account, lower NNT indicates that treatment is more effective [48]. To place our observations in BCSs within a broader medical context, the NNT of cancer survivors for CBT-I treatment insomnia and prevention of depression is 3 [43], substantially lower compared to 21 for statins—a widely used medication to prevent another myocardial infarction. Development, testing, and broad dissemination of a selective depression prevention strategies in cancer survivors is required.

These data also provide a compelling rationale for routine screening for depression during the cancer treatment trajectory and into the survivorship period. Even in the absence of insomnia, BCSs are at elevated risk of depression. Importantly, those who experience depressive symptoms and a depressive disorder have a high risk of premature deaths, especially in older adult samples [10, 11]. Moreover, as the population ages and the numbers of cancer survivors grow dramatically, depression is projected to increase by 2030 to a position of the greatest contributor to illness burden [49], contributing to other medical comorbidity such as cardiovascular disease which can occur years after cancer treatments [50]. Whereas team-based, collaborative care approaches including medical oncologists, primary care physicians, mental health can effectively treat depression [51–53], only about 30%–35% of patients with major depressive disorder achieve remission using current pharmacologic treatments [54], leaving over two-thirds of patients with depression burden intact [55, 56]. Together, these findings emphasize the importance of depression monitoring with delivery of treatment or interventions for indicated depression prevention.

Among the prospective studies that have examined the contribution of insomnia to risk of depression, almost no studies have examined the potential mechanisms [57, 58]. However, it is now well established that insomnia leads to inflammatory activation [59], which can be reversed following insomnia treatment [60–63]. Such increases in inflammation may be due to the effects of insomnia to increase sympathetic outflow; sympathetic signaling of the β-adrenergic receptor leads to the activation inflammatory transcriptional pathways, expression of inflammatory genes, as well as to in vivo production of inflammatory cytokines and increases in systemic inflammation [59]. In contrast, the direct effect of sympathetic activation on depression has only mixed support [64–67]. In turn, experimental studies have found that inflammatory activation induces depressive symptoms [68, 69] and predicts incident depression [69, 70]. Given that BCSs show increases in systemic inflammation [71], secondary analysis will examine whether inflammation mediates the association between insomnia and depression. Additionally, systemic inflammation is a plausible biological pathway linking depression to cancer survival [72].

### Limitations

Certain limitations must be considered. Although selection bias is a concern, BCSs enrolled in this study had similar demographic and clinical characteristics as overall breast cancer patients in the KPSC integrated healthcare system. Because patients with current or recent history of depression were excluded, it is possible that our results may not be generalizable to a broader group of older patients who suffer from chronic depression. Second, insomnia was ascertained by the ISI, rather than a diagnostic interview. However, this screening strategy could be broadly used in oncology settings to identify insomnia and depression risk. Finally, in the older adult CC, insomnia did not increase depression risk. Nevertheless, we have found that insomnia increases the risk of recurrent, but not incident, depression in community-dwelling older adults [22]. Given that both BCSs and CC were identified from the KPSC membership with insurance, health surveillance may have attenuated rates insomnia and depression risk. Nonetheless, this health plan reflects population diversity in terms of race, ethnicity, and other sociodemographics [73].

## Conclusions

Older adult, long-term BCSs show an increased likelihood of depression as compared to those without cancer. Moreover, subthreshold insomnia defined by screening with the 7-item ISI and a score >8 was found to exaggerate the risk of incident and recurrent depression in BCSs. Given that insomnia is a priority target for selective depression prevention [24, 74–76], direct evidence of the effectiveness of insomnia treatment in the prevention of depression in BCSs is needed.

## Data Availability

Deidentified individual-level patient data that support the findings of this study are available from the corresponding author upon request. A detailed proposal for how the data will be used is required, and we will assess applications on a case-by-case basis. All proposals should be submitted to the corresponding author.
